# Comparison of Adolescent Depression Screening Using Orally Administered Versus Written Self-Report Scores

**DOI:** 10.1177/21501319251374583

**Published:** 2025-09-15

**Authors:** Erich K. Batra, Wen-Jan Tuan, Deepa Sekhar, Ritika Merai, Tesia Shi, Benjamin N. Fogel

**Affiliations:** 1 Penn State College of Medicine, Hershey, PA, USA; 2 National Institute of Mental Health, Bethesda, MD, USA

**Keywords:** depression, pediatrics, primary care, mood disorder, prevention

## Abstract

**Objective:**

The objective of our retrospective study was to evaluate differences between screening methods (oral administration versus written self-report) for adolescent depression in an outpatient setting

**Study Design:**

We analyzed data from 4075 well-child check (WCC) visits from adolescents (ages 12-18 years) at an academic medical center from January 2022 through December 2023. We evaluated the outcomes of depression screening questions from both those asked by staff (oral administration) and those filled out on paper by the patient (written self-report). A composite score of 3 or greater (out of 6) indicates a positive screen for depression. Logistic regression was used to assess for the likelihood of discrepancy between scores.

**Results:**

Of the 4518 WCC visits analyzed, 3380 (75%) had completed data for both the orally administered and the written screenings. The scores were equal in 2563 (76%) visits; the written score was greater in 766 (22.6%) visits and the oral score was greater in 51 (1.5%) visits. The screen was positive for depression in 232 (6.8%) visits for the written self-report compared with 66 (2.0%) from the oral administration. Logistic regression analyses showed likelihood of score differences were higher in older age, female gender, Hispanic race/ethnicity, and those with public insurance.

**Conclusion:**

This preliminary pilot study shows that there are score differences in depression screening when administered orally by staff versus self-reported in writing, and scores may be higher on the written self-report screening. Limitations of this study include slight differences in the wording of the questions and lack of rigorous protocol guidelines.

## Introduction

Depression in adolescents is a significant public health concern. In 2019, 15.8% of adolescents aged 12 to 17 years reported a major depressive episode in the past year, with rates increasing post COVID-19 pandemic.^1,2^ Screening tools can be used to detect depression before symptoms may be apparent to healthcare providers, with the goal of implementing a treatment or prevention strategy to prevent future morbidity. Given that primary care often serves as the first point-of-entry for mental health services, depression screening in this setting is important for identification and treatment. The American Academy of Pediatrics (AAP) Guidelines for Adolescent Depression in Primary Care recommends that “all adolescent patients ages 12 years and older should be screened annually for depression (ie, major depressive disorder [MDD] or other depressive disorders) with an evidenced-based self-report screening tool either on paper or electronically.”^3^ This was supported by a recommendation by the US Preventive Services Task Force to screen all adolescents aged 12 to 18 years for major depressive disorder.^4^ Prior research has highlighted depression screening for adolescent patients to be acceptable with patients and families and feasible to implement in medical settings.^5^ Screening in primary care has also shown to improve early diagnosis and connect at-risk adolescents to needed treatment and care.^6^

As electronic health records (EHRs) evolve, many offices have tried to eliminate the need for paper questionnaires by recording responses directly in the EHR. While there are several validated screening tools for adolescent depression, their administration falls into 1 of 2 main categories: orally administered by health care staff or self-administered by the patient on paper or electronically. The administration method of the screening tool varies by settings and available resources and may affect the responses that patients give. Studies that review depression screens in adults that were self-administered compared to oral administration by a health professional have yielded conflicting results. One study in a primary care setting found similar screening results when comparing self-administrated depression screenings to oral screenings via telephone.^7^ Two other studies, one with adult sickle cell patients and the other with geriatric patients, found higher scores with self-report screens compared to screens orally administered by staff.^8,9^ These results could have been due to patients feeling reduced stigma when completing self-administered questionnaires and a perception of increased privacy. Prior research on other health topics have also compared oral versus written self-reported questionnaire. For example, 1 study showed a significant increase in food insecurity reports when changing from oral to written questionnaires.^10^ Although written administration can be more time efficient^11^ and ensures standardization of question wording, it may miss non-verbal cues,^12^ which are important for clinical judgement. Other studies have found that electronic mental health screening can facilitate increased disclosure and has high acceptability among patients.^12-16^

There is a lack of studies comparing the results of depression screening administered orally by health care staff versus self-reported by patient on paper. Additionally, prior research has primarily focused on adult populations, and there are currently no studies the authors could identify that focused specifically on depression screening in adolescent populations. Adolescent stakeholders have highlighted the importance of such a comparison and acknowledged the value of providers addressing depressive symptoms during the well-child visit.^17^ The aim of this study was to compare results of oral administration by staff versus written self-reported administration of depression screening questions in adolescents. An exploratory aim was to examine whether the results varied by various demographic characteristics (sex, age at visit, race/ethnicity, and insurance type).

## Methods

This naturalistic research study utilized a retrospective design using data from an enterprise EHR data warehouse. The study population consisted of all patients aged 12 to 18 years who had well-child check visits at 1 pediatric clinic of a regional academic health center in central Pennsylvania between January 2022 and December 2023. A total of 2545 patients were included in the final analysis. The study was approved by the health center’s institutional review board.

## Measures

### Patient Health Questionnaire-2 (PHQ-2)

The PHQ-2 is a brief, 2 question, validated depression screener^18,19^ that inquires about symptoms in the last 2 weeks. The 2 items on the PHQ-2 in our EHR that are asked by the nursing staff are “Feeling down or depressed?” and “Little interest or pleasure in doing things?” Each response is numerically scored where “not at all” is scored as 0, “several days” as 1, “more than half the days” as 2, and “nearly every day” as 3. A score of 3 or greater is a positive screen for depression.

### Patient Health Questionnaire-A (PHQ-A)

The Patient Health Questionnaire – 9 (PHQ-9) is a validated depression screening tool that is commonly used in adults, and has also been validated in adolescents.^20^ It consists of 9 questions that query MDD signs and symptoms in the prior 2 weeks. The PHQ-A, an adolescent-specific version of the PHQ-9, includes slight question wording changes to be more appropriate for young people (ie, PHQ-A: feeling down, depressed, irritable, or hopeless vs PHQ-9: feeling down, depressed, or hopeless). To ensure the data is comparable to the PHQ-2, only the first 2 questions of the PHQ-A were used in the analysis of this study which inquired how often the patient has been bothered by the following symptoms in the last 2 weeks: “Feeling down, depressed, irritable, or hopeless?” and “Little interest or pleasure in doing things?”^21^ Although the first 2 questions of the PHQ-A are not typically scored in isolation, for the purposes of this study, a composite score of 3 or greater (range = 0-6) on these first 2 questions is considered a positive screen for depression in order to allow for direct comparisons with the PHQ-2.

During the check-in process of each WCC visit at this clinic, patients were asked the PHQ-2 depression questions orally by nursing staff and also completed the PHQ-A questionnaire on paper. Both responses were entered into the EHR. Because of this redundancy, the clinic presented an opportunity to compare responses of oral versus self-reported written administration of depression screening questions.

### Analytic Plan

Patient demographics including sex, age at visit, race/ethnicity (Hispanic, non-Hispanic White, non-Hispanic Black, Asian, Other), and insurance type (commercial, public, and uninsured) are reported descriptively. Missing data analysis was performed using the Chi-square test to assess differences in the distribution of the population characteristics between visits with both types of screeners completed and those with missing measures.

Proportion differences in categorical variables were evaluated using the Chi-square test. To understand if patient characteristics were associated with the likelihood of discrepancy between orally administered and self-reported written screens, we performed a logistic regression using the binary outcome measure of 0 (scores were equivalent) and 1 (scores were different). The adjusted odds ratios (aORs) and 95% confidence intervals (CIs) for each baseline variable were calculated to assess the likelihood of each outcome measure. The significance level was set at a 2-tailed *P* < .05. Variance Inflation factor was computed to measure the inflation in the variances of the regression parameter estimates due to collinearities that exist among the predictors.

## Results

Data were analyzed from 4518 WCC visits of patient ages 12 to 18 years old between January 2022 and December 2023. Out of the total number of visits, 4075 (90%) had a completed PHQ-2 (oral administration by staff), 3739 (83%) had a completed PHQ-A (written self-report) and 3380 out of 4518 visits (75%) had completed both a PHQ-2 and a PHQ-A. The 3380 visits represented 2545 unique patients.

Patient characteristics of those patients who filled out both are summarized in Table 1. The study population had an almost equal distribution of males and females, with an average age of 14.6 years. Majority of participants identified as non-Hispanic White (63.5%) and had commercial insurance (69.4%). Analysis of the missing scores did not show statistical differences in population characteristics between included visits (those with both screeners completed) and excluded visits (those with any screener missing).

**Table 1.** Summary Statistics by Patient Characteristics (N = 3380).

Characteristics	Statistics	*p*-value
Age at visit, n (%)		<0.01
12	589 (17.4)	
13	561 (16.6)	
14	567 (16.8)	
15	506 (15.0)	
16	495 (14.6)	
17	407 (12.0)	
18	255 (7.5)	
Sex, n (%)		0.45
Female	1668 (49.3)	
Male	1712 (50.7)	
Race/ethnicity, n (%)		<0.01
Hispanic	345 (10.2)	
White, non-Hispanic	2147 (63.5)	
Black, non-Hispanic	188 (5.6)	
Asian, non-Hispanic	117 (3.5)	
Other, non-Hispanic	583 (17.2)	
Payer category, n (%)		<0.01
Commercial	2345 (69.4)	
Public	915 (27.1)	
Uninsured	120 (3.6)	

Abbreviation: n, number of patients.

Of the 3380 visits when both screens were completed, there was a 3.5 times greater screen positive rate for depression on the written self-report screen (232 visits, 6.8%) compared with the oral administration 1 (66 visits, 2.0%)

When both scores were present, the majority of scores were equal (76%). There were 766 occurrences (22.6%) when the PHQ-A (written) had a higher score than the PHQ-2 (oral), and 51 occurrences (1.5%) when the PHQ-2 (oral) had a higher score than the PHQ-A (written). Logistic regression results (Table 2) of the whole sample demonstrated greater odds of a score discrepancy for older adolescents (aOR = 1.08 [95% CI = 1.03, 1.12], *P* < .001) and females (aOR = 1.66 [95% CI = 1.41, 1.95], *P* < .001). Compared to non-Hispanic whites, Hispanic adolescents (aOR = 1.44 [95% CI = 1.11, 1.87], *P* < .01) and adolescents in the Other race group (aOR = 1.29 [95% CI = 1.04, 1.60], *P* = .02) were more likely to show discrepancies between PHQ-2 and PHQ-A scores. Publicly insured adolescents were 1.6 times (aOR = 1.62 [95% CI = 1.35, 1.95] *P* < .001) more likely to have different scores than adolescents with commercial insurance.

**Table 2.** Logistic Regression of Likelihood of Having Different PHQ-2 and PHQ-A Scores Among Adolescent Patients.

Characteristics	Adjusted odds ratio (95% CI)	*p*-value
Age at visit	1.08 (1.031,1.123)	<.01
Female (ref: male)	1.66 (1.409,1.946)	<.01
Race/Ethnicity (ref: non-Hispanic White)		<.01
Hispanic	1.44 (1.105,1.868)	<.01
Black, non-Hispanic	1.28 (0.905,1.797)	.16
Asian, non-Hispanic	1.03 (0.657,1.608)	.91
Other, non-Hispanic	1.29 (1.040,1.595)	.02
Payer category (ref: commercial)		<.01
Public	1.62 (1.349,1.949)	<.01
Uninsured	1.35 (0.880,2.057)	.17

Abbreviation: CI, conference internals.

## Discussion

Screening for depression at pediatric WCC visits is an important component of adolescent care. The majority of responses in this study (approximately 76%) had equal scores for oral administration versus written self-report. When they were not equivalent, the written self-report was typically higher, which may be indicative of greater comfort disclosing depression signs/symptoms with this modality. This has important implications both for future screening and treatment, as higher reported scores could lead to increased recognition of depression and earlier opportunity for intervention.

When scores differed, we saw significant differences in the 2 screening methods. Our study showed a 3.5 times greater screen positive rate on the self-reported on paper administration compared to the oral administration. The PHQ-A instrument has a total of 9 questions and would not typically be scored using only the first 2 questions in isolation. However, by comparing the first 2 questions of the PHQ-A with the oral administration of the PHQ-2, our findings suggest that there may be a difference in patient responses between the 2 methods. This correlates with the findings in 2 prior adult studies showing a higher depression screen from self-report than when asked by staff.^8,9^

The logistic regression analysis showed that older adolescents, female patients, Hispanic patients, and those with public insurance had a significantly higher likelihood (*P* < .01) of having a higher score on the self-reported screen than the orally administered one. One reason could be stigma, as minority patients may not want to admit verbally about depression symptoms to staff and a written self-report may be viewed as more private.^22,23^ This is an area worthy of exploration for future research.

The PHQ-A was a self-administered questionnaire, which may have influenced the accuracy of responses based on age and gender. Older adolescents may have been better able to understand and accurately report their depressive symptoms compared to younger adolescents. It is unclear why females were more likely to have differences between the 2 methods.

Differences between the PHQ-2 and PHQ-A screening methods were also observed across payer categories. Compared to individuals with commercial insurance, those with public insurance were 1.6 times more likely to have higher scores on the PHQ-A than on the PHQ-2. Exact reasons for this are unclear from our study.

The findings from this preliminary study indicate there may be a higher screen positive rate on a written screen compared to when the screening is orally administered by the staff and invites further studies with adolescents to examine reasons for this difference. It may be that the adolescents feel more pressure to disclose when the questions are asked directly by staff. Similarly, written self-reports may provide more privacy and reduce the likelihood of a parent/guardian viewing the responses compared to oral administration.

When screening tools are administered outside of a controlled study environment, the way questions are asked will differ based on who is asking. One advantage of using a written screening instrument is that it can minimize variability in wording, body language, or intonation in how staff may ask the questions orally. It is crucial to train staff to use the exact verbiage on the tool to avoid leading questions and reduce bias. At the same time, we know that adolescents want to feel their provider is listening and reflecting on what they share and not just processing their complaints.^24^ One potential advantage of oral administration is that the individual administering the screening can assess the patient’s body language, intonation and other verbal and non-verbal cues. In a busy primary care practice, this would typically not be accomplished during a busy check-in process by a nurse, but ideally could lead to a more robust discussion with the provider.

There are some important limitations of our study, which also reflect challenges in real-world clinical care. First, the patient is typically handed the PHQ-A on paper upon check-in, and then the nursing staff asks the PHQ-2 questions when rooming the patient. We were not able to determine if this process was always completed in this order, and it is possible that the order in which the forms are completed could affect results. Second, while it is recommended that the adolescent completes the written form independently, it was not possible to determine if there was any parent or guardian input or viewing of the forms. Third, there is a difference in wording between the first question of the PHQ-2 (oral administration) in our EMR, which states “down or depressed,” and the PHQ-A (written report) which states “feeling down, depressed, irritable, or hopeless?” It is unknown how much, if at all, having the additional terms “irritable” or “hopeless” on the written PHQ-A affected the results. Finally, it was not possible for the research team to verify that each nurse asked questions exactly as written during oral administration. In a prospective study, these questions should be given in an alternating order, without parental input, and worded exactly the same.

This preliminary study demonstrates that adolescents had more positive screens for depression on a written self-report than oral administration by staff. However, it is important to note that this study does not comment on which administration method is more “accurate” and is not meant to be a definitive statement on how depression screening tools should be administered. To determine this, prospective studies with follow-up psychological evaluation are needed to determine how many individuals would be diagnosed with depression. Future studies are also needed to determine whether screening tools that are validated for 1 method of administration are also valid when administered in other ways and whether method of administration affects the results of tests.

This study provides a real-world example of differences in outcome when screening questions are administered orally versus self-reported on paper. We found higher scores from written self-reported adolescent depression screening when compared with screening questions that were orally administered by nursing staff. These results support further research on the development of tool-specific and evidence-based guidance for the administration of mental health screening tools.
